# Efficacy and Safety of Mizoribine for the Treatment of Refractory Nephrotic Syndrome: Protocol for a Multicenter, Controlled, Open-label, Randomized Controlled Trial

**DOI:** 10.2196/46101

**Published:** 2023-06-16

**Authors:** Zheyi Dong, Jianhui Zhou, Zhonggao Xu, Zhaohui Ni, Yani He, Hongli Lin, Gengru Jiang, Xuefeng Sun, Li Zhang, Xiangmei Chen

**Affiliations:** 1 Department of Nephrology First Medical Center of Chinese People's Liberation Army General Hospital Beijing China; 2 Nephrology Institute of the Chinese People's Liberation Army First Medical Center of Chinese People's Liberation Army General Hospital Beijing China; 3 State Key Laboratory of Kidney Diseases First Medical Center of Chinese People's Liberation Army General Hospital Beijing China; 4 National Clinical Research Center for Kidney Diseases First Medical Center of Chinese People's Liberation Army General Hospital Beijing China; 5 Beijing Key Laboratory of Kidney Disease Research First Medical Center of Chinese People's Liberation Army General Hospital Beijing China; 6 The First Hospital of Jilin University Changchun China; 7 Ren Ji Hospital Shanghai Jiao Tong University School of Medicine Shanghai China; 8 Daping Hospital Army Medical University Chongqing China; 9 Key Laboratory of Kidney Disease The First Affiliated Hospital of Dalian Medical University Dalian China; 10 The Center for the Transformation Medicine of Kidney Disease The First Affiliated Hospital of Dalian Medical University Dalian China; 11 XinHua Hospital School of Medicine Shanghai Jiao Tong University Shanghai China

**Keywords:** refractory nephrotic syndrome, mizoribine, cyclophosphamide

## Abstract

**Background:**

Nephrotic syndrome that is resistant to steroid therapy is termed refractory nephrotic syndrome (RNS), a condition that is associated with an increased risk of end-stage renal disease. Immunosuppressants are used to treat RNS; however, prolonged use may lead to significant adverse effects. Mizoribine (MZR) is a novel agent used in long-term immunosuppressive therapy, which has few adverse effects, but data on its long-term use in patients with RNS are unavailable.

**Objective:**

We propose a trial to examine the efficacy and safety of MZR compared with cyclophosphamide (CYC) in Chinese adult patients with RNS.

**Methods:**

This is a multicenter, randomized, controlled interventional study with a screening phase (1 week) and a treatment phase (52 weeks). This study has been reviewed and approved by the Medical Ethics Committees of all 34 medical centers that are participating. Patients with RNS consent to participation, and are enrolled and randomized to an MZR group or a CYC group (1:1 ratio), with each group receiving tapering doses of oral corticosteroids. Participants are assessed for adverse effects, and laboratory results are collected at 8 visits during the treatment phase (weeks 4, 8, 12, 16, 20, 32, 44, and 52 [exit visit]). Participants are able to withdraw voluntarily, and investigators are required to remove patients when there are safety concerns or deviations from the protocol.

**Results:**

The study started in November 2014 and was completed in March 2019. A total of 239 participants from 34 hospitals in China have been enrolled. Data analysis has been completed. The results are being finalized by the Center for Drug Evaluation.

**Conclusions:**

This study examines the safety and efficacy of MZR as a long-term treatment approach for Chinese adults with RNS. It is the longest lasting and largest randomized controlled trial to examine MZR in Chinese patients. The results can help determine whether RNS should be considered as an additional indication for MZR treatment in China.

**Trial Registration:**

ClinicalTrials.gov NCT02257697; https://clinicaltrials.gov/ct2/show/NCT02257697

**International Registered Report Identifier (IRRID):**

RR1-10.2196/46101

## Introduction

Patients with nephrotic syndrome (NS) have the following classic triad of symptoms: heavy proteinuria, edema, and hypoalbuminemia [1]. Additional complications in these patients, such as venous thromboembolism, infection, and, less commonly, acute renal failure, can lead to catastrophic outcomes [2]. Patients with NS who are resistant to or dependent on corticosteroids for more than 4 to 6 months are considered to have refractory nephrotic syndrome (RNS), also referred to as steroid-resistant nephrotic syndrome, although RNS is a complex condition and there have been differences in how this term is used [3,4]. Almost all patients with NS who have proteinuria eventually progress to end-stage renal disease (ESRD) [3,5]. Because prednisone therapy often fails to provide complete remission of proteinuria, immunosuppressants may also be used to prevent or treat RNS [6]. Although several immunosuppressants (eg, cyclophosphamide [CYC], azathioprine, cyclosporine, and rituximab) can effectively treat proteinuria in patients with RNS [7,8], prolonged use of these drugs can lead to severe adverse effects (SAEs), such as sexual dysfunction, tumorigenesis, and renal dysfunction, and patients may also experience recurrence after discontinuation [9]. A novel immunosuppressant therapy that causes fewer side effects and that is suitable for long-term therapy is therefore needed for the treatment of RNS.

Mizoribine (MZR) was originally isolated from an ascomycete, *Eupenicillium brefeldianum*. Initial studies reported it had weak activity against *Candida albicans*, and subsequent studies confirmed it suppressed lymphocyte proliferation, and humoral and cellular immunity [10-12]. Other studies had examined its immunosuppressive effects and its use with other immunosuppressants for preventing rejection after kidney transplantation. Japan has approved MZR for the treatment of lupus nephritis, rheumatoid arthritis, and primary NS. Previous studies showed that MZR led to fewer SAEs, such as myelosuppression and hepatotoxicity, than other immunosuppressants [10,12]. Several clinical trials confirmed the clinical efficacy of MZR when it was used as a long-term therapy for the treatment of certain autoimmune diseases, such as lupus nephritis, rheumatoid arthritis, and primary NS [10,12]. Kawasaki et al [10] also reported that MZR was superior to corticosteroids in reducing proteinuria in patients with RNS and that the adverse effects (AEs) were similar in MZR and control groups. However, due to its relatively low efficacy, there is only limited data regarding the effect of MZR in patients with RNS, especially in adults with complex etiologies.

We propose a randomized, multicenter, controlled study to evaluate the efficacy and safety of MZR compared with CYC for the treatment of adults with RNS.

## Methods

### Ethical Considerations

This clinical trial is performed in accordance with the principles in the latest versions of the Declaration of Helsinki and Chinese Good Clinical Practice, and applicable clinical trial regulations. This study has been approved by the Medical Ethics Committee of the Chinese People’s Liberation Army General Hospital on September 15, 2014 (C2014-042-01). Documents describing the study protocol, informed consent, safety monitoring policy, AEs, and management policies have been reviewed and approved with minor modifications by the Medical Ethics Committee. Approval of this trial has also been granted by appropriate Ethics Committees and the Center for Drug Evaluation (CTR20130299). Written informed consent for publication has been obtained from all participants.

### Overview

This multicenter, randomized, controlled, open-label clinical study assesses the efficacy and safety of MZR in comparison with CYC for the treatment of RNS in adult patients. This clinical trial is performed at 34 centers in 27 cities in China.

The principal investigators at the 34 different sites receive patients from inpatient and outpatient settings who are referred and might be good candidates for participation. All potential candidates are carefully screened according to the inclusion and exclusion criteria. Those who qualify are approached by investigators for study enrollment. Study details, potential benefits, and potential risks of participating are explained in detail to all patients and to family members (if present), and all questions are answered. Patients who express full understanding and are willing to participate sign the informed consent documents, and these are co-signed by the investigators who recruit them. Each eligible and enrolled participant is offered US $15 as compensation for transportation expenses. The participants pay for all concomitant medications. The sponsor pays for the study medications and tests. The clinic visits and laboratory procedures are free of charge. The medical care needed for any AEs is also covered by the sponsor. All data are deidentified to protect patient confidentiality. The protected health information of all participants is entered by authorized staff or investigators into the electronic case report form in an electronic database that is managed by Tigermed Data Management. Any access to the system records or change of data in the electronic case report form after the original entry is automatically recorded, and each patient’s protected health information is secured. All paper documents, such as consent forms, are collected and locked in the investigators’ offices.

### Key Study Objectives

The primary objective is to compare the therapeutic effect of MZR with CYC (the current standard therapy) for the treatment of RNS by analyzing overall and total remission rates in the different groups.

The secondary objective is to evaluate the efficacy and safety profiles of MZR for the treatment of RNS, including but not limited to clinical remission rate, progression rate, treatment failure rate (not achieving partial remission), 24-h urine protein, serum albumin, serum creatine, and estimated glomerular filtration rate (eGFR) as endpoints.

The exploratory objective is to evaluate the changes in high-sensitivity C-reactive protein (hs-CRP) levels in patients who receive MZR.

### Trial Design

**Figure 1:**
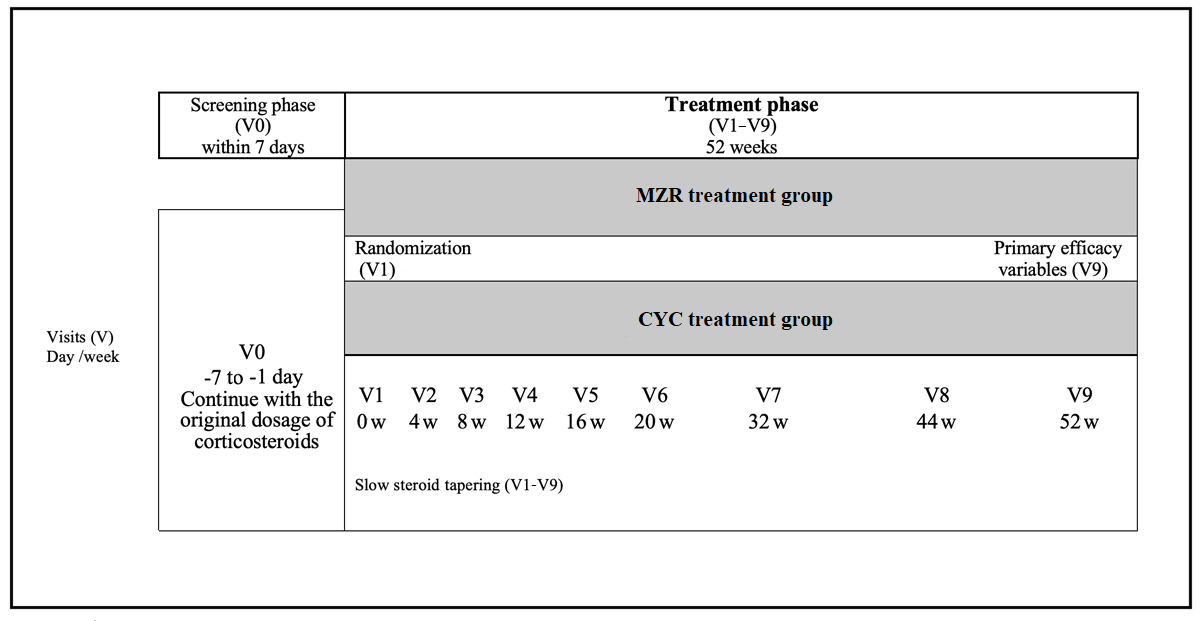
Study timeline. Randomization is performed on day 1 of V1. CYC: cyclophosphamide; MZR: mizoribine; V: visit.

The specific aim of this study is to assess the hypothesis that MZR is not inferior to CYC for the treatment of RNS. This study consists of a 1-week screening phase followed by a 52-week treatment phase (Figure 1). Signed informed consent agreements are collected upon patient enrolment. The investigators have identified 238 eligible participants during the screening phase (visit 0; V0) according to the inclusion and exclusion criteria. Baseline information, including medical records, are obtained at this time. All enrolled participants are then randomized to an MZR group or a CYC group (1:1 ratio).

**Figure 2:**
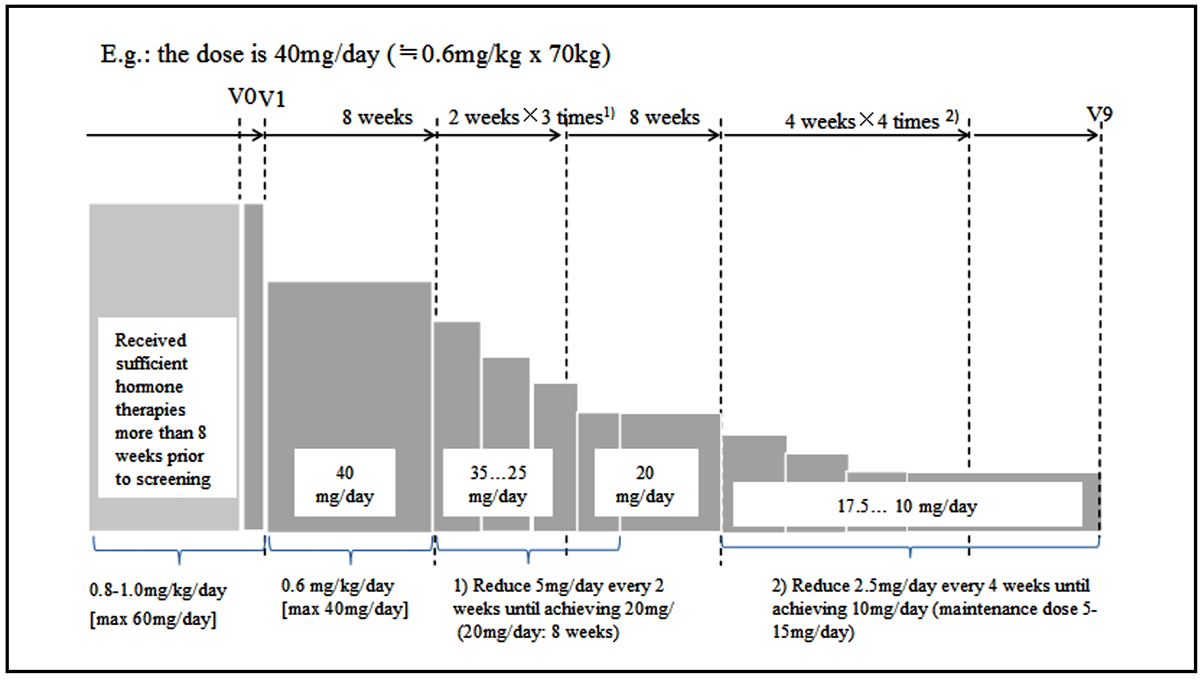
Plan for corticosteroid dose tapering. This example is for a dose of 40 mg/day (0.6 mg/kg x 70 kg). V: visit.

There are 9 visits (V1-V9) by each participant during the treatment phase, during which examinations are performed, AEs are evaluated, and samples are collected for laboratory tests performed at a central laboratory (Figure 2). Each visit is within 7 days of the scheduled date. All participants from both groups receive tapering oral corticosteroid therapy during the entire treatment phase (Figure 2). Patients in both groups receive specific doses of the treatment drugs in addition to corticosteroids. AEs and symptoms are closely monitored and documented. Participants are able to withdraw voluntarily, and investigators are required to remove participants when there is any concern regarding safety, according to the withdrawal protocol.

### Eligibility Criteria

#### Inclusion Criteria

Patients are eligible for inclusion if they meet all of the following criteria: (1) Documentation of a confirmed diagnosis of NS; (2) Receipt of a renal biopsy within 1 year prior to screening, with a confirmed pathologic diagnosis of minimal change disease, immunoglobulin (Ig) A nephropathy, mesangioproliferative glomerulonephritis, membranous nephropathy (MN), or focal segmental glomerulosclerosis (FSGS), so that a planned subgroup analysis can be performed for these different pathological classifications; (3) Receipt of an adequate dosage of corticosteroids (0.8-1.0 mg/kg/day prednisolone or equivalent dose of another corticosteroid for more than 8 weeks [more than 12 weeks for FSGS]) based on the pathologic diagnosis mentioned in “inclusion criterion 2” before screening; (4) 24-h urine protein of 2.0 g or more at screening; (5) Male or female gender and age from 18 to 70 years upon signing the informed consent agreement; (6) Body weight between 40 and 80 kg at screening; and (7) Capacity and willingness to sign the informed consent document.

#### Exclusion Criteria

Patients are excluded if they have any of the following conditions: (1) NS caused by primary diseases other than those listed in “inclusion criterion 2” (eg, membranoproliferative glomerulonephritis); (2) NS from a secondary cause (eg, diabetic nephropathy, anaphylactic purpura nephritis, lupus nephritis, type B hepatitis-related nephritis, and renal amyloidosis); (3) History of allergy to MZR or CYC; (4) Receipt of an accumulated dosage of more than 3 g CYC during the 1 year before screening; (5) Receipt of immunosuppressants or any Chinese traditional medicine with immunosuppressive effects within 30 days before screening; (6) Receipt of disallowed therapeutic medications within 30 days before screening; (7) Receipt of plasma exchange therapy or immunoadsorption therapy within 30 days before screening; (8) Need for pentostatin or a live vaccine (not including the flu vaccine); (9) Receipt of renal replacement therapy; (10) Receipt of kidney transplantation; (11) Malignancy; (12) Severe hypertension (systolic blood pressure >160 mmHg or diastolic blood pressure >100 mmHg) that is not effectively controlled; (13) Low white blood cell count (<3×10^9^/L); (14) Elevated serum creatinine (SCr; >176.8 μmol/L [2 mg/dL]); (15) Elevated levels of liver enzymes (aspartate transaminase or alanine transaminase 3 times higher than the upper limit of normal); (16) Hepatitis B, hepatitis C, or HIV infection; (17) Other infections based on chest computed tomography; (18) Unsuitability to participate according to the investigators because of uncontrolled diabetes, central nervous system lupus, lupus encephalopathy, active psychosis, osteonecrosis of the femoral head, fulminant hepatitis, peptic ulcer, etc; (19) Pregnancy, breast feeding, or planning to become pregnant; and (20) Any other disease that may affect the evaluation of medication efficacy and safety.

#### Premature Withdrawal Criteria

Any participant who has signed the informed consent form and has initiated the study can be withdrawn prior to the last visit for any of the following reasons: (1) Withdrawal by the participant (the participant decides to withdraw during the screening or interventional phase); (2) Screening failure (the participant does not satisfy all inclusion criteria or meets one of the exclusion criteria at V0 and V1 [randomization visit]); (3) Protocol violation (the participant has a serious violation of the inclusion or exclusion criteria after the first dose of medication, uses a prohibited medication or prohibited therapy before the last planned visit, or has another major violation); (4) Pregnancy; (5) Poor compliance with the study medication (the participant has poor compliance with the protocol, including refusal of follow-up, laboratory tests, or treatments); (6) Investigators’ decision (the investigators decide the participant has a medical condition, including those listed in the exclusion criteria, or has personal circumstances during the interventional phase that might cause substantial risk on continuing treatment; or the professional evaluations and decisions of the investigators do not allow the participant to follow the study protocol); (7) AE (the participant has any clinical AE or SAE according to the investigators’ professional judgment; if an AE is not considered to be clinically related to the study medications or if the benefits of continuing treatment outweigh the risks, the treatment is continued at the discretion of the investigators; a low white blood cell count [≤3×10^9^/L] or an elevated aspartate transaminase or alanine transaminase level [>5 times the upper limit of normal] necessitates withdrawal); (8) Loss to follow-up (the participant fails to visit the study site for any reason before the last planned visit or by the end of the study); (9) Reaching an endpoint (progression to ESRD or doubling of SCr) at any time during the study; and (10) Others (difficulty for the participant to continue the study based on judgment of the principal investigator or co-investigators).

### Randomization Strategy

Eligible participants are randomized to an MZR group or a CYC group in a 1:1 ratio using the Interactive Web Response System (IWRS) on day 1. A unique subject number is assigned to each participant. Stratification is according to pathological classification. When a participant who fails the screening is deemed eligible after rescreening by the investigators, the participant is allowed to enter the randomization before the end of enrollment.

### Interventions

#### Study Medications

##### MZR

Participants in the MZR group start receiving MZR at V1. The oral dose is 50 mg per tablet, 3 times per day, corresponding to a total daily dose of 150 mg. Administration continues until V9 (Figure 1).

##### CYC

Participants in the CYC group start receiving CYC at V1. The dose is calculated as 0.5 to 1.0 g/m^2^ body surface area, with a maximum dose of 1.0 g/day. Administration occurs from V1 to V8 (Figure 1).

##### Corticosteroids

All participants receive oral corticosteroid therapy (0.8 to 1.0 mg/kg/day prednisolone or an equivalent dose of methylprednisolone) during the screening phase. A dose of 0.6 mg/kg/day for 8 weeks begins at V1, and the maximum daily dosage is 40 mg. Starting from V3, the corticosteroid dose is tapered gradually, with a dose reduction of 5 mg every 2 weeks until the daily dose is 20 mg/day (if tolerated). Prednisolone (20 mg/day) or an equivalent dose of methylprednisolone is continued for 8 weeks. The dose is tapered by 2.5 mg every 4 weeks until the maintenance dose of 5 to 15 mg/day is attained at V9 (Figure 2).

#### Screening Period (V0)

The investigators obtain written informed consent from all participants prior to any intervention, and all participants are registered in the IWRS. During the screening period, participants are screened by the investigators for collection of relevant information. This information includes medical history, medications and previous therapies, and AEs (if any). Height, body weight, and vital signs are measured, and a 12-lead resting electrocardiogram (ECG) is obtained. Blood is collected for laboratory tests, including complete blood count (CBC), biochemistry, eGFR, hepatitis B surface antigen, hepatitis C virus antibody, HIV, IgG, and hs-CRP. A routine urine test for 24-h protein is performed. Women of childbearing age are tested for pregnancy (Multimedia Appendix 1).

#### Treatment Period (V1-V9)

##### V1 (Randomization Visit)

Each participant’s laboratory results, physical examination results, medical history, medication history, and pathology reports are reviewed for evaluation of eligibility according to the inclusion and exclusion criteria before enrollment and randomization. Participants are then registered in the IWRS. If they qualify and are enrolled, appropriate doses of the study medication are dispensed, and the investigators provide information on how to take the medication and appropriate storage. The investigators also emphasize the importance of compliance, and request the return of all unused medications (Multimedia Appendix 1).

##### V2-V9

During this phase, all women of childbearing age return to the study center for pregnancy tests whenever they think there is a possibility of pregnancy. All participants are logged in to the IWRS when they visit the study center. Appropriate doses of the study medications are dispensed, and the investigators reinforce the importance of appropriate dosing and storage. The importance of compliance and the return of unused medications is also addressed, and detailed information regarding AEs is recorded. Pregnancy tests are routinely performed in all women of childbearing age. Laboratory tests, including CBC, biochemistry, eGFR, routine urine test, pregnancy test for women of childbearing age (V6 and V9), IgG, 24-h urine protein, and hs-CRP, are performed. Body weight and vital signs are measured at each visit, and a second ECG is performed at V9 (Multimedia Appendix 1).

#### Unscheduled Visit

Study participants who come to the study site for an unscheduled visit are documented as such. The reason for any unscheduled visit is documented in detail. Laboratory tests are performed if necessary, and vital signs, body weight, ECG, etc are assessed as needed. An urgent or emergent situation, such as potential pregnancy or disease progression, is evaluated and addressed by the study investigators.

#### Premature Withdrawal From the Study (End of Study)

Any participant who is prematurely withdrawn from the study after confirmation receives an examination within 1 week from the withdrawal date, and this event is documented. The examination consists of necessary tests and other procedures that are recommended by the investigators and accepted by the participant.

The study design and items collected at all visits are summarized in Multimedia Appendix 1.

### Outcome Measures

#### Primary Efficacy Variables

The primary efficacy variable is the total remission rate (%) and is based on data collected from each group at V9. The total remission rate is calculated as the sum of the complete remission rate (%) and the partial remission rate (%). Complete remission is defined as 24-h urine protein less than 0.3 g, and partial remission is defined as 24-h urine protein decline of more than 50% from baseline (V0) and value of less than 3.5 g.

#### Secondary Efficacy Variables

The secondary efficacy variables are the complete remission rate and partial remission rate of each group at V9; remission rate at V3, V6, V7, V8, and V9; treatment failure, defined as not achieving partial remission; and 24-h urine protein, serum albumin, SCr, eGFR (using the Chronic Kidney Disease Epidemiology Collaboration [CKD-EPI] formula), and blood urea nitrogen values. The endpoint events are progression to ESRD or doubling of the SCr relative to baseline.

#### Exploratory Variables

The exploratory variables are the hs-CRP levels in each group at the different visits.

### Safety Assessment

The safety of participants is actively monitored during the study. All events, including unplanned pregnancy, are documented and properly managed. The reportable events include but are not limited to SAEs (including death), pregnancy, important treatment-related AEs (granulocytopenia, infections, hemorrhagic cystitis, liver function impairment, hyperuricemia, malignancy, amenorrhea, alopecia, nausea, and vomiting), and AEs leading to study discontinuation.

Safety is monitored by asking participants about symptoms, and by assessment of physical examination results and laboratory test results (CBC, blood biochemistry, IgG, hs-CRP, pregnancy test, routine urine test, body weight, vital signs [blood pressure, pulse, and body temperature], chest computed tomography, and 12-lead ECG).

### Statistical Plans

The sample size calculation is based on a noninferiority comparison of the MZR and CYC groups regarding the total remission rate after treatment, and is to be calculated using SAS (SAS Institute). Studies of RNS assumed the total remission rate in each group was 60% [13,14] and the risk ratio of MZR to CYC was 1.0. Thus, we include 119 participants in each group based on the following assumptions: total remission rate of 60% for each group, noninferiority difference ratio of 0.674, power of 80%, 1-sided significance level of 2.5%, and dropout rate of 20%.

The full analysis set (FAS) includes all participants who are randomized and treated with at least one dose of the study medication and who have received at least one posttreatment efficacy assessment. The per-protocol set (PPS) includes all FAS participants who have no major protocol violation. The safety set includes all randomized participants who receive at least one dose of the study medication and have at least one safety assessment.

The primary and secondary efficacy outcomes are assessed and analyzed. A noninferiority test of total remission is performed in the PPS population by comparing the 2 groups at V9, and relative risk ratios and 2-sided 95% CIs are calculated. A secondary analysis of the primary efficacy variable is performed in the PPS at the end of the study and in the FAS at V9. The number of participants who achieve total remission in the PPS is calculated in each group at each visit. The secondary efficacy variables are analyzed using descriptive statistics. The safety assessment is performed in the safety set population. The incidences of AEs in the 2 groups are compared. Subgroup analysis is performed as necessary, such as exploratory outcome analysis using hs-CRP and other variables.

## Results

The study began in November 2014 and was completed in March 2019. A total of 239 participants from 34 hospitals in China were enrolled. Data analysis has been completed. The results are being finalized by the Center for Drug Evaluation.

## Discussion

NS is caused by abnormally increased protein leakage from the kidneys into the urine [15]. In adults, proteinuria greater than 3.5 g/1.73 m^2^ per day is considered nephrotic. The incidence of NS in the general population is about 1 per 100,000 [16]. The manifestations of NS are more complex in adults than children, because adults experience a wider variety of primary and systemic diseases, making management more difficult.

MN, FSGS, and minimal change disease are the most common causes of idiopathic RNS in adults [17-20]. Diabetes mellitus is the most common cause of secondary NS, and other causes include systemic lupus erythematosus, hepatitis B or C, amyloidosis, and multiple myeloma [2]. The pathogenesis of secondary NS differs from that of primary NS in that treatments for primary NS focus on the primary disease. Steroid hormone regimens and immunosuppression strategies also differ for primary and secondary NS. Thus, we exclude patients with secondary NS from this study. A potential limitation of this study is that the pathologic types of RNS may be concentrated in a few common pathologic types in Chinese patients, such as MN.

RNS remains poorly understood but is currently considered immune mediated because many patients respond to immunosuppressive drugs [21]. The immune mechanisms underlying the pathophysiology of RNS may include systemic T- or B-cell dysfunction [22]. In particular, cytokines produced by T cells are thought to act on the slit diaphragms of renal podocytes and increase protein permeability [23]. Alkylating agents, such as CYC, can inhibit T cells and B cells, have a wide range of applications in RNS, and are often used as positive controls in clinical studies. However, CYC and other traditional alkylating agents can cause myelosuppression, infertility, bladder cancer, leukemia, skin cancer, and hemorrhagic cystitis [24]. The optimal regimen to be used for immunosuppression in patients with RNS is uncertain, and many clinical trials are evaluating different therapeutic schedules for patients with different pathological types of RNS. Calcineurin inhibitors mainly inhibit T cells and can induce remission, but there is nearly a 50% risk of relapse after cessation of calcineurin inhibitors in patients with idiopathic MN [25]. Rituximab is a B-cell–depleting agent, and when used alone, it can induce disease remission and can cause few SAEs [26].

MZR was originally isolated as an antibiotic agent that had activity against *C. albicans*, and subsequent studies reported it had strong immunosuppressive activity in various animal experimental models [11]. MZR is an imidazole nucleoside, and its metabolite, MZ-5-P, selectively inhibits inosine monophosphate synthetase and guanosine monophosphate synthetase, resulting in the complete inhibition of guanine nucleotide synthesis without incorporation into nucleotides. MZR also has selective inhibitory effects on inosine 5-monophosphate dehydrogenase, an enzyme that functions in de novo purine nucleotide synthesis. MZR thus suppresses cell-mediated and humoral immune responses by suppressing T- and B-lymphocyte proliferation via the inhibition of guanosine monophosphate synthesis [27,28]. This mechanism is similar to that of CYC, but MZR is associated with fewer AEs. Two previous small head-to-head trials of specific agents compared the relative effectiveness and safety profiles of MZR and CYC in adult patients with MN [29,30]. Both studies showed that MZR reduced albuminuria and had a good safety profile.

Accordingly, this clinical trial evaluates the efficacy and safety of MZR in comparison with CYC for the treatment of RNS in Chinese adults with glomerular diseases. It is a multicenter, randomized, controlled trial, and we assess the efficacy of MZR based on changes in total proteinuria, eGFR, and AEs.

There is no universal treatment regimen used for adults with primary NS, although corticosteroids are beneficial in pediatric patients. Adults with NS usually receive conservative management and steroid treatment when conservative therapies fail [16,31]. However, the long-term use of immunosuppressants for the treatment of RNS may lead to SAEs, with unclear benefits. MZR appears to cause less severe AEs and provides good outcomes when used to treat other immune-related conditions, such as lupus nephritis, disorders after kidney transplantation, and primary nephritic syndrome [10,12,32,33]. However, there is currently insufficient evidence regarding its efficacy and safety for the treatment of RNS.

The proposed study examines the efficacy and safety of MZR as a long-term treatment approach for adults with RNS. It is so far the largest randomized controlled trial to examine MZR in China. The trial lasted about 1 year, and frequent follow-ups have been used to monitor efficacy and AEs. Confirmation of the efficacy and safety of MZR could lead to the improved management of adults with RNS and could improve our understanding of the pathology of RNS caused by different renal diseases.

## Appendix

Multimedia Appendix 1 Study design and items collected at different visits.

## Abbreviations

AE: adverse effect
CBC: complete blood count
CYC: cyclophosphamide
ECG: electrocardiogram
eGFR: estimated glomerular filtration rate
ESRD: end-stage renal disease
FAS: full analysis set
FSGS: focal segmental glomerulosclerosis
hs-CRP: high-sensitivity C-reactive protein
Ig: immunoglobulin
IWRS: Interactive Web Response System
MN: membranous nephropathy
MZR: mizoribine
NS: nephrotic syndrome
PPS: per-protocol set
RNS: refractory nephrotic syndrome
SAE: serious adverse effect
SCr: serum creatinine

## Data Availability

The data sets generated during this study are not publicly available due to the incomplete clinical analysis but will be made available by the corresponding author on reasonable request.
