# Supplementary material for: Efficacy and Safety of Mizoribine for the Treatment of Refractory Nephrotic Syndrome: Protocol for a Multicenter, Controlled, Open-label, Randomized Controlled Trial
Source: JMIR Res Protoc. 2023 Jun 16;12:e46101. doi: 10.2196/46101 (PMC10337463; doi:10.2196/46101)
Supplement: Multimedia Appendix 1 [file resprot_v12i1e46101_app1.docx]

**Multimedia Appendix 1.** Study design and items collected at different visits.

| Category and items for assessment | | | Screening phase | | Treatment phase | | | | | | | | | | | | | | |
| --- | --- | --- | --- | --- | --- | --- | --- | --- | --- | --- | --- | --- | --- | --- | --- | --- | --- | --- | --- |
|  | | | Visit (V) 0 (−7 d to −1 d) | | V1 (0 wk; randomization) | V2 (4 wk ± 7 d) | V3 (8 wk ± 7 d) | V4 (12 wk ± 7 d) | V5 (16 wk ± 7 d) | V6 (20 wk ± 7 d) | V7 (32 wk ± 7 d) | V8 (44 wk ± 7 d) | | V9 (52 wk ± 7 d) | | UNS^a^ | | EOS^b^ | |
|  | | |  | |  |  |  |  |  |  |  |  | |  | |  | |  | |
| **Screening criteria** | | |  | |  |  |  |  |  |  |  |  | |  | |  | |  | |
|  | Informed consent | ✓ | |  | |  |  |  |  |  |  | |  | |  | |  | |  |
|  | Inclusion criteria/exclusion criteria^c^ | ✓ | | ✓ | |  |  |  |  |  |  | |  | |  | |  | |  |
| **Medical history** | | |  | |  |  |  |  |  |  |  |  | |  | |  | |  | |
|  | Demographic/medical history^d^ | ✓ | |  | |  |  |  |  |  |  | |  | |  | |  | |  |
|  | Medical history of nephrotic syndrome^e^ | ✓ | |  | |  |  |  |  |  |  | |  | |  | |  | |  |
|  | Renal biopsy and pathological classification^f^ | ✓ | |  | |  |  |  |  |  |  | |  | |  | |  | |  |
| **Patient management** | | |  | |  |  |  |  |  |  |  |  | |  | |  | |  | |
|  | IWRS^g^ log in | ✓ | | ✓ | | ✓ | ✓ | ✓ | ✓ | ✓ | ✓ | | ✓ | |  | | ✓ | |  |
|  | Randomization |  | | ✓ | |  |  |  |  |  |  | |  | |  | |  | |  |
| **Medication and therapy** | | |  | |  |  |  |  |  |  |  |  | |  | |  | |  | |
|  | Study drug dispense (cyclophosphamide) |  | | ✓ | | ✓ | ✓ | ✓ | ✓ | ✓ | ✓ | | ✓ | |  | |  | |  |
|  | Study drug dispense (mizoribine) |  | | ✓ | | ✓ | ✓ | ✓ | ✓ | ✓ | ✓ | | ✓ | |  | |  | |  |
|  | Return of the study drug^h^ |  | | ✓ | | ✓ | ✓ | ✓ | ✓ | ✓ | ✓ | | ✓ | | ✓ | |  | | ✓ |
|  | Compliance of the study drug |  | | ✓ | | ✓ | ✓ | ✓ | ✓ | ✓ | ✓ | | ✓ | | ✓ | |  | | ✓ |
|  | Corticosteroid therapy^i^ | ✓ | | ✓ | | ✓ | ✓ | ✓ | ✓ | ✓ | ✓ | | ✓ | | ✓ | | ✓ | | ✓ |
|  | Concomitant medication and therapy^j^ | ✓ | | ✓ | | ✓ | ✓ | ✓ | ✓ | ✓ | ✓ | | ✓ | | ✓ | | ✓ | | ✓ |
| **Adverse effects** | | |  | |  |  |  |  |  |  |  |  | |  | |  | |  | |
|  | AE^k^ | ✓ | | ✓ | | ✓ | ✓ | ✓ | ✓ | ✓ | ✓ | | ✓ | | ✓ | | ✓ | | ✓ |
|  | SAE^l^, pregnancy | ✓ | | ✓ | | ✓ | ✓ | ✓ | ✓ | ✓ | ✓ | | ✓ | | ✓ | | ✓ | | ✓ |
| **Laboratory** | | |  | |  |  |  |  |  |  |  |  | |  | |  | |  | |
|  | Biochemistry^m^ | ✓ | |  | | ✓ | ✓ | ✓ | ✓ | ✓ | ✓ | | ✓ | | ✓ | | ✓ | | ✓ |
|  | Routine CBC^n^ | ✓ | |  | | ✓ | ✓ | ✓ | ✓ | ✓ | ✓ | | ✓ | | ✓ | | ✓ | | ✓ |
|  | Routine urine test^o^ | ✓ | |  | | ✓ | ✓ | ✓ | ✓ | ✓ | ✓ | | ✓ | | ✓ | | ✓ | | ✓ |
|  | 24-h urinary protein^p^ | ✓ | |  | | ✓ | ✓ | ✓ | ✓ | ✓ | ✓ | | ✓ | | ✓ | | ✓ | | ✓ |
|  | hs-CRP^q^ | ✓ | |  | | ✓ | ✓ | ✓ | ✓ | ✓ | ✓ | | ✓ | | ✓ | |  | | ✓ |
|  | Pregnancy test (female)^r^ | ✓ | |  | |  |  |  |  | ✓ |  | |  | | ✓ | | ✓^s^ | | ✓ |
|  | HBsAg^t^, HCVAb^u^, HIV^v^ | ✓ | |  | |  |  |  |  |  |  | |  | |  | |  | |  |
|  | IgG^w^ | ✓ | |  | | ✓ | ✓ | ✓ | ✓ | ✓ | ✓ | | ✓ | | ✓ | | ✓ | | ✓ |
| **Vital signs** | | |  | |  |  |  |  |  |  |  |  | |  | |  | |  | |
|  | Height | ✓ | |  | |  |  |  |  |  |  | |  | |  | |  | |  |
|  | Body weight | ✓ | |  | | ✓ | ✓ | ✓ | ✓ | ✓ | ✓ | | ✓ | | ✓ | | ✓ | | ✓ |
|  | Vital signs (blood pressure, pulse, and temperature) | ✓ | |  | | ✓ | ✓ | ✓ | ✓ | ✓ | ✓ | | ✓ | | ✓ | | ✓ | | ✓ |
| **ECG^x^** | | |  | |  |  |  |  |  |  |  |  | |  | |  | |  | |
|  | 12-lead resting ECG | ✓ | |  | |  |  |  |  |  |  | |  | | ✓ | | ✓ | | ✓ |
| **CT^y^** | | |  | |  |  |  |  |  |  |  |  | |  | |  | |  | |
|  | Chest CT | ✓ | |  | |  |  |  |  |  |  | |  | |  | | ✓ | | ✓ |

^a^UNS (unscheduled visit) should be arranged if an adverse effects (eg, infection) is suspected between 2 planned visits.

^b^EOS (end of study) with a final examination when the participant discontinues or drops out.

^c^Laboratory tests at V0 for inclusion/exclusion screening.

^d^Race, gender, date of birth, and medical history.

^e^Medical history of nephrotic syndrome (date and age at diagnosis).

^f^Renal biopsy and pathological classification within the year prior to V0.

^g^IWRS: Interactive Web Response System.

^h^Onsite destruction of used cyclophosphamide vials.

^i^Planning details as in Figure 2.

^j^Concomitant medications and therapies, including angiotensin converting enzyme inhibitor and angiotensin receptor blocker treatment. Prohibited medications and treatments include immunosuppressive agents and Chinese traditional medicine with immunosuppressive effects other than the study drug, live vaccines (not including the flu vaccine), pentostatin, other investigational drugs, plasma exchange therapy, immunoadsorption therapy, and corticosteroid pulse therapy.

^k^Adverse effects (AEs) and protocol-defined important AEs.

^l^SAE: severe adverse effect.

^m^Biochemistry from the central laboratory (alanine transaminase, aspartate transaminase, total bilirubin, total protein, albumin, uric acid, total cholesterol, triglycerides, glucose, serum creatinine, blood urea nitrogen, and estimated glomerular filtration rate).

^n^Routine CBC (complete blood count) from the central laboratory (hemoglobin, hematocrit, white blood cells, red blood cells, and platelet count).

^o^Routine urine test from the central laboratory (dipstick test: urine protein and urine glucose).

^p^24-h urine protein from the central laboratory; the investigators should determine whether a patient requires hospitalization for collection of 24-h urine to determine urinary protein; 24-h urine protein results from V0 are planned to be used if further testing is unsuitable.

^q^hs-CRP (high-sensitivity C-reactive protein) from the central laboratory.

^r^Pregnancy test for women of childbearing age from the central laboratory.

^s^Pregnancy test for women of childbearing potential as needed.

^t^HBsAg (hepatitis B surface antigen) test from the central laboratory.

^u^HCVAb (hepatitis C virus antibody) test from the central laboratory.

^v^HIV test from the central laboratory.

^w^IgG (immunoglobulin G) test from the central laboratory.

^x^ECG: electrocardiogram.

^y^CT: computed tomography.
